# Fully automated segmentation of the cervical cord from T1-weighted MRI using *PropSeg*: Application to multiple sclerosis^☆^

**DOI:** 10.1016/j.nicl.2015.11.001

**Published:** 2015-11-10

**Authors:** Marios C. Yiannakas, Ahmed M. Mustafa, Benjamin De Leener, Hugh Kearney, Carmen Tur, Daniel R. Altmann, Floriana De Angelis, Domenico Plantone, Olga Ciccarelli, David H. Miller, Julien Cohen-Adad, Claudia A.M. Gandini Wheeler-Kingshott

**Affiliations:** a NMR Research Unit, Queen Square MS Centre, Department of Neuroinflammation, UCL Institute of Neurology, London, UK; b Institute of Biomedical Engineering, Polytechnique Montreal, Montreal, QC, Canada; c Department of Medical Statistics, London School of Hygiene and Tropical Medicine, London, UK; d Functional Neuroimaging Unit, CRIUGM, Université de Montréal, Montreal, QC, Canada; e Brain Connectivity Center, C. Mondino National Neurological Institute, Pavia, Italy

**Keywords:** Magnetic resonance imaging, Image segmentation, Cord cross-sectional area, Grey matter, White matter

## Abstract

Spinal cord (SC) atrophy, i.e. a reduction in the SC cross-sectional area (CSA) over time, can be measured by means of image segmentation using magnetic resonance imaging (MRI). However, segmentation methods have been limited by factors relating to reproducibility or sensitivity to change. The purpose of this study was to evaluate a fully automated SC segmentation method (*PropSeg*), and compare this to a semi-automated active surface (*AS*) method, in healthy controls (HC) and people with multiple sclerosis (MS). MRI data from 120 people were retrospectively analysed; 26 HC, 21 with clinically isolated syndrome, 26 relapsing remitting MS, 26 primary and 21 secondary progressive MS. MRI data from 40 people returning after one year were also analysed. CSA measurements were obtained within the cervical SC. Reproducibility of the measurements was assessed using the intraclass correlation coefficient (ICC). A comparison between mean CSA changes obtained with the two methods over time was performed using multivariate structural equation regression models. Associations between CSA measures and clinical scores were investigated using linear regression models. Compared to the *AS* method, the reproducibility of CSA measurements obtained with *PropSeg* was high, both in patients and in HC, with ICC > 0.98 in all cases. There was no significant difference between *PropSeg* and *AS* in terms of detecting change over time. Furthermore, *PropSeg* provided measures that correlated with physical disability, similar to the *AS* method. *PropSeg* is a time-efficient and reliable segmentation method, which requires no manual intervention, and may facilitate large multi-centre neuroprotective trials in progressive MS.

## Highlights

• *PropSeg* is a fully automated segmentation method to measure cord atrophy in MS.
• *PropSeg* offers comparable results to a widely used semi-automated method.
• *PropSeg* is available for immediate clinical utility.

## 1 Introduction

Neuropathological and magnetic resonance imaging (MRI) studies have demonstrated the involvement of the spinal cord (SC) in multiple sclerosis (MS); neurodegeneration in the SC is thought to represent the main pathological substrate of irreversible locomotor disability (Abdel-Aziz et al., 2015, DeLuca et al., 2006, Ganter et al., 1999). In particular, SC MRI has provided indirect evidence of axonal degeneration by quantifying atrophy, i.e. a reduction in SC cross-sectional area (CSA) over time, with correlations identified between measures of CSA and physical disability (Kearney et al., 2015b, Lin et al., 2004, Losseff et al., 1996). Such associations support the notion that reliable CSA estimation over time could be a plausible endpoint for clinical trials for neuroprotection in MS (Kearney et al., 2014a), and a number of exploratory studies have been reported in the literature (Kalkers et al., 2002, Leary et al., 2003).

Previous methods used for measuring CSA have been variable in terms of their reproducibility and sensitivity to small change, and all of them require some degree of operator input (Coulon et al., 2002, Horsfield et al., 2010, Kawahara et al., 2013, Kidd et al., 1993, McIntosh et al., 2011). Typically, intra- and inter-observer reproducibility is assessed from repeated measurements by estimating the coefficient of variation (COV); the currently established semi-automated active surface (*AS*) method offers intra- and inter-observer COV values of 0.44% and 1.07%, respectively (Horsfield et al., 2010). More recently, investigators have aimed to develop fully automated segmentation methods, which may minimize user-bias and significantly reduce the image processing time (Asman et al., 2014, Chen et al., 2013, Koh et al., 2011).

However, the variety of image acquisitions, the types of image contrast and variability of the field of view (FOV) required for each specific application, make it particularly challenging for each individual method to simultaneously account for so many variables. A fully automated method, called *PropSeg*, which accounts for such variability, has been recently developed (De Leener et al., 2014). *PropSeg* is based on an iterative propagation of a deformable model with adaptive contrast mechanisms and offers fast and reliable measurements of the cord CSA in a matter of seconds, as demonstrated in a pilot study of healthy volunteers and people with spinal cord injury (De Leener et al., 2014); importantly, the method has been reported to work when using T1-, T2- and T2*-weighted acquisitions and at any level of the spinal cord.

In this study we evaluate *PropSeg*, as compared to the widely used semi-automated *AS* method (Horsfield et al., 2010), in a large cohort of healthy controls and people with MS, in order to test the following hypotheses:

(i) *PropSeg* provides reproducible CSA measurements in the cervical SC.
(ii) A reduction in CSA in the cervical SC, seen longitudinally in MS, can be reliably measured with *PropSeg*.
(iii) There are associations between cervical SC CSA measures derived by *PropSeg* and clinical scores in MS.

## 2 Materials and methods

### 2.1 Study participants

MRI data from 120 people were retrospectively analysed; 26 healthy controls (HC), 21 people with clinically isolated syndrome (CIS), 26 relapsing remitting (RR) MS, 21 secondary progressive (SP) MS and 26 primary progressive (PP) MS. The inclusion criteria for the CIS cohort, and the criteria used for MS diagnosis and MS subgroup classification, have been reported previously (Kearney et al., 2014b, Kearney et al., 2015a).

All people with CIS and MS had Expanded Disability Status Scale (EDSS) (Kurtzke, 1983) and Multiple Sclerosis Functional Composite (MSFC) score (Fischer et al., 1999) determined by the same neurostatus certified assessor. Z-scores for the 25-foot timed walk test (TWT), 9-hole peg test (HPT) and 3 s paced auditory serial addition test B (PASAT) were calculated using published normative values. For those participants who could not perform the TWT and HPT, an arbitrary value of 180 s or 300 s was assigned to that test, respectively. In addition, the American Spinal Injury Association (ASIA) motor (m) and sensory (s) scores (Maynard et al., 1997) were recorded for all participants. All clinical assessments were performed immediately before the MRI study. Demographic and clinical characteristics are summarised in Table 1.

A total of 40 people returned for follow-up assessment, with MRI and clinical assessments repeated at the second visit; 10 HC (4 female (F), mean age (SD): 43.4 (8.9) years), 10 RRMS (6 F, 40.5 (9) years), 10 SPMS (4 F, 56.3 (5.9) years) and 10 PPMS (2 F, 56.2 (8.5) years). The mean (SD) follow-up visit for the HC was (14 (5.2) months), RRMS (24 (3.74) months), SPMS (16.3 (3.6) months) and PPMS (14.8 (4.9) months).

Informed written consent was obtained from each study participant prior to inclusion in the study. The study received approval from the local Institutional Ethics Committee.

### 2.2 MRI acquisition protocol

Imaging was performed using a 3 T Philips Achieva MRI system with RF dual-transmit technology (Philips Medical Systems, Best, Netherlands) and the manufacturer's product 16-channel neurovascular coil.

The whole cervical cord was imaged using a magnetization-prepared 3D T1-weighted acquisition (with isotropic voxel size of 1 mm^3^) in the sagittal plane with FOV = 256 × 256 mm^2^, matrix size = 256 × 256, TR = 8 ms, TE = 3.7 ms, TI = 860 ms (using linear *k*-space profile order), SENSE = 2 in the anterior–posterior direction and TFE factor of 205; the scan time for the acquisition was 6:30 min.

### 2.3 Image analysis

The 3D T1-weighted volume obtained from each study participant was processed using both the active surface (*AS*) (Horsfield et al., 2010) (Jim 6.0_019; http://www.xinapse.com/) and *PropSeg* (De Leener et al., 2014) (Spinal Cord Toolbox version 1.0; https://sourceforge.net/projects/spinalcordtoolbox/) segmentation methods in two different ways, which provide the CSA at C2/C3 and between C2 and C5, respectively: i) by reformatting the original sagittal volume in the axial plane and extracting 15 contiguous 1 mm thick slices orthogonal to the longitudinal axis of the cervical cord centred at the C2/C3 level – this was done using the multi-planar reconstruction option available within Jim 6.0 that allows to manually position the handle of the reformatted volume orthogonal to the longitudinal axis of the cervical cord centred at the C2/C3; the volume was subsequently resampled using sinc interpolation along the slice direction – and ii) by using the axial reformatted volume obtained from i), only this time processing a larger number of axial slices to cover the section of the cervical cord from the top of C2 to the base of C5 vertebral body as previously reported (Horsfield et al., 2010). The rationale for selecting and processing these two segments of the cervical cord in this study is based on previously published methods in MS, which were shown to offer reproducible atrophy measurements and/or were used to investigate the possibility that specific levels of the cervical cord were particularly sensitive to MS-related atrophy (Horsfield et al., 2010, Kearney et al., 2014a, Losseff et al., 1996, Rocca et al., 2011).

#### 2.3.1 AS analysis method

**Fig. 1:**
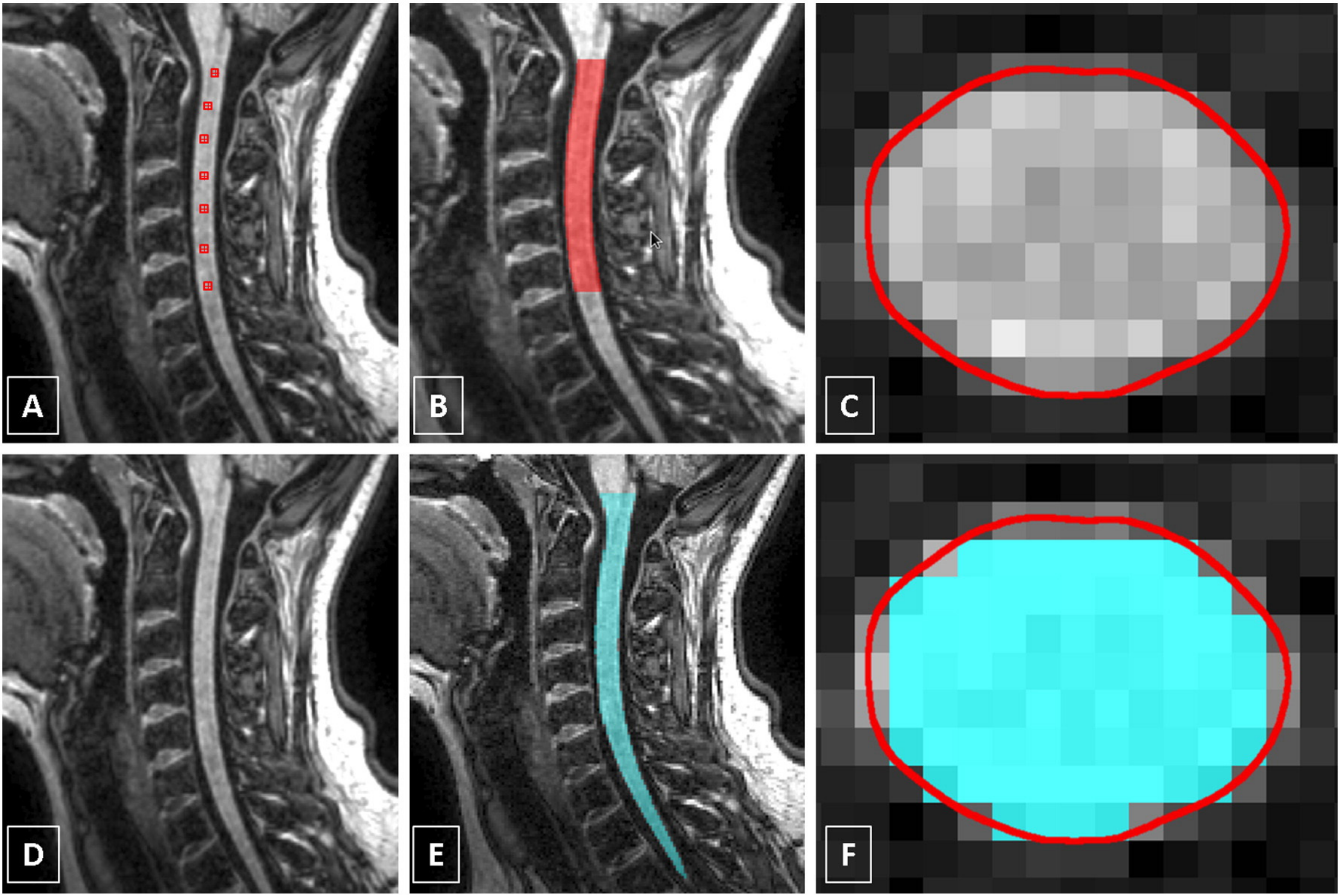
(A–C) Segmentation of the cervical cord using the active surface (*AS*) method; seed points are manually positioned in the centre of the cord to cover the C2/C5 level; also shown is an example of a single axial reformatted slice through the C2/C3 intervertebral disc showing the cord contour identified using the *AS* method (contour shown in red). (D–F) Segmentation of the cervical cord using the *PropSeg* method with the sagittal volume (contour shown in cyan); also shown is the same axial reformatted slice through the C2/C3 intervertebral disc showing the cord contour identified using both the *PropSeg* (contour shown in cyan) and *AS* methods (contour shown in red) for comparison. (For interpretation of the references to colour in this figure legend, the reader is referred to the web version of this article.)

Using the *AS* method, each scan was processed by a single rater (MY) as follows: a seed point was first placed in the centre of the cord on the most superior axial slice in which the odontoid process of the axis (C2) was still visible. The next seed point was placed in the centre of the cord on the slice that passed through the inferior border of C5. Starting at C5 and moving superiorly, a seed point was placed in the centre of the cord on every tenth slice until the seed point at the top of C2 was reached (Horsfield et al., 2010) (see Fig. 1 A–C). In this way, the boundary of the cord on all slices from C2 to C5 was identified and 15 slices corresponding to the C2/C3 level were subsequently processed for method i) and all the slices processed for method ii).

#### 2.3.2 PropSeg analysis method

Using the *PropSeg* method, all 3D T1-weighted volumes were processed in their original form (sagittal plane) taking only a few minutes in total, simply by specifying the directory storing all the data. The processed volumes containing the binary mask of the whole cervical SC were then reformatted in the axial plane to match the processing of the *AS* method i.e. by extracting the equivalent slices as per i) and ii) described earlier. Fig. 1 D–F shows an example of the result obtained using *PropSeg* with the original sagittal volume and an example of a single axial reformatted slice through the C2/C3 intervertebral disc showing the cord contour identified using both *PropSeg* and *AS* segmentation methods for comparison.

Whilst for i) an equal number of slices was processed in all cases, the number of slices processed in ii) was not always the same due to anatomical variability; for this reason, CSA measurements were normalized by the number of slices as previously suggested (Healy et al., 2012).

### 2.4 Statistics

Statistical analyses were performed using Stata 13.1 (Stata Corp.).

#### 2.4.1 Reproducibility assessment

Since the *PropSeg* method inherently outputs the same result each time the same scan is analysed, i.e. intra- and inter-observer reproducibility COV = 0%, the most appropriate test of reproducibility in this case was related to the ability of each segmentation method to obtain near-identical measurements when a number of the study participants underwent the same MRI examination twice (i.e. ‘scan–rescan’ assessment). For this purpose, 8 healthy controls (6 males, mean age 33.5, SD 6.7) and 8 people with MS (5 females, mean age 43.3, SD 11.3, 4 RRMS, 4 SPMS) had the scan twice after being removed from the scanner and repositioned between the scans during the same visit.

For the assessment of scan–rescan reproducibility, the intraclass correlation coefficient (ICC) was calculated and subsequently 1-ICC was reported; 1-ICC provides an estimate of the fraction of variability due to measurement error (within-subject) over the total variation, i.e. biological variation (between-subject) and within-subject variation (Bartlett and Frost, 2008). 95% confidence intervals (CI) and p-values were obtained using bias-corrected and accelerated non-parametric bootstrap with 1000 replicates.

#### 2.4.2 Change in CSA over time and effect size calculations

Mean changes in cord CSA over one year were investigated for each participant group (apart from CIS), each cervical SC segment and each segmentation method. For each group of participants and for each cervical SC segment, a formal comparison between mean CSA changes obtained with the two segmentation methods was performed using multivariate (bivariate) structural equation regression models; in this context these essentially fit two regression models simultaneously, allowing the comparison, across models, of relevant coefficients.

To assess the potential usefulness of the CSA measure to detect change, the change ratio (CR), the ratio of the mean of within-subject changes/standard deviation (SD) of within-subject changes, was calculated for each segmentation method, each cord segment, and each group. This is because the sensitivity to change, or the power of a method, is related not to the absolute magnitude of the change but to the change relative to the SD of changes. To assess the potential sensitivity to patient pathology, for each patient group, effect size (ES) was calculated as the difference between the mean CSA change in that patient group and the mean CSA change in the control group, divided by the SD of the change in the patient group; again the magnitude of the difference relative to SD is crucial. For both CR and ES measures, higher values indicated a greater sensitivity and power of the MRI measure to detect change or difference. MRI measures with large CR denote that the individuals of a group show a homogeneously large amount of change over time relative to the ‘noise’. Similarly, MRI measures with large ES would reflect a large and homogeneous difference between the change in a given group of patients and that in controls.

#### 2.4.3 Associations between CSA measures and clinical scores

In order to investigate and compare associations between the clinical and the two segmentation MRI measures, each baseline clinical variable (for each cervical cord segment) was used as the response (dependent) variable in linear regression models, with the following explanatory variables: i) baseline *AS*-derived CSA; ii) baseline *PropSeg* CSA; iii) both *AS* and *PropSeg* baseline CSA. For each clinical variable, a comparison was made between the R-square of the model in i) and that of the model in ii). Models obtained in iii) were used to assess the comparative potential of the two segmentation methods to explain the variability of the clinical variable. Similar models were performed using one-year MRI and one-year clinical measures, and using baseline MRI and one year clinical measures. In this exploratory work, a number of statistical tests were performed. However, these were in order to examine several null hypotheses as opposed to a single one; for this reason adjustment for multiple comparisons was not made (Perneger, 1998). Significance level was set at 5%.

## 3 Results

**Fig. 2:**
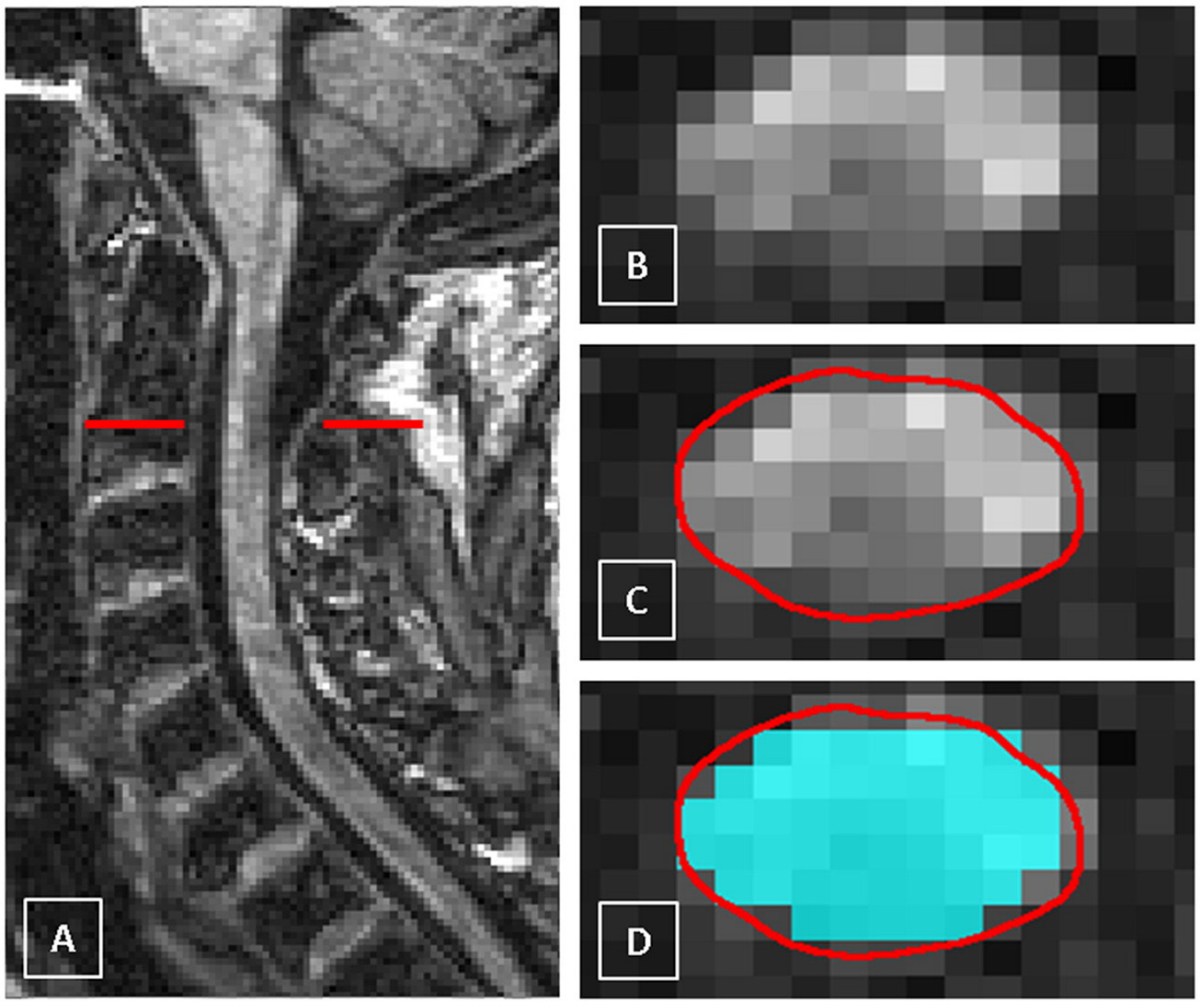
A) The cervical cord in the sagittal plane showing a multiple sclerosis (MS) lesion (hypointense) at the C2 level in a case of secondary progressive MS (SPMS), B) axial slice through the level of the lesion at the C2 level, C) the cord contour identified using the *AS* method (contour shown in red) and D) the same axial slice through the MS lesion at C2 showing the cord contour identified using both the *PropSeg* (contour shown in cyan) and *AS* methods (contour shown in red) for comparison. (For interpretation of the references to colour in this figure legend, the reader is referred to the web version of this article.)

Representative mean (SD) CSA measurements obtained at each cervical SC level with each segmentation method and for each participant group at baseline are shown in Table 1. Out of 160 scans processed in total, *PropSeg* failed to correctly segment the cord only in 3 cases (1 healthy control and 2 RRMS), and these cases were manually processed by inserting seed points in the centre of the cord prior to the segmentation; the presence of MS lesions had no obvious effect on the performance of the segmentation method (see Fig. 2). Segmentation of the cord using the *AS* method was successful in all cases.

### 3.1 Reproducibility assessment

In the HC group, the estimated ICC values for the C2/C3 and C2/C5 levels were very similar using both segmentation methods. In the patient group, the estimated ICC values were slightly higher using the *AS* method than *PropSeg*, for both cervical cord levels. Nevertheless, the estimated ICC values were always above 0.98 (Table 2).

### 3.2 Change in CSA over time and effect size calculations

For the C2/C3 level, mean changes measured using *AS* and *PropSeg* methods were not significantly different for any of the groups, except for a borderline evidence of a higher (negative) change in RRMS using *PropSeg* than *AS* (p = 0.0425).

For the C2/C5 level, in controls there was no evidence of any of the changes over time, with either *PropSeg* or *AS*, being different from zero. No differences were observed between methods for the other groups.

As regards the CR of one-year change in CSA, apart from PPMS, CR was slightly higher using *PropSeg* than *AS* and for both segments of the cervical SC (Table 3); CSA reduction was greater in patients than controls, although not statistically significant.

The ES, for the C2/C3 level was better using *PropSeg* than *AS*, apart from the PPMS group. Instead, for the C2/C5 level, *PropSeg* was worse than *AS* in all patient groups (Table 4).

### 3.3 Associations between CSA measures and clinical scores

Univariable models showed that baseline CSA measures for both segments of the SC were significantly associated with baseline clinical variables, for both segmentation methods (Table 5). At one-year follow-up, CSA measures were only significantly associated with ASIA-m and ASIA-s, for both cervical SC segments (Table 6). Baseline CSA measures for both segments predicted ASIA-m scores at one-year follow-up, for *PropSeg* and *AS* methods (for C2/C3: p = 0.001 and p = 0.003, respectively; for C2/C5: p < 0.001 and p = 0.001, respectively) (Table 7).

Multiple regression models showed that baseline CSA measures obtained with *PropSeg* method were better at explaining the variability of the EDSS and ASIA-m (for the C2/C3 segment: p = 0.085 and p = 0.022, respectively; for the C2/C5 segment: p = 0.049 and p = 0.048). Additionally, baseline *PropSeg* measures at C2/C3 explained better the variability of ASIA-s (p = 0.020) than *AS* method. At one-year follow-up, there was no evidence that any of the methods was better than the other at explaining the variability of any of the clinical measures. As regards prediction analyses, there was borderline evidence of the *PropSeg* method (C2/C5 measures at baseline) explaining better than the *AS* method (also at baseline, C2/C5 measures) the variability of ASIA-m at one-year follow-up.

## 4 Discussion

This is the first study to apply a fully automated method (*PropSeg*) of spinal cord area measurement to people with MS. The results of this study demonstrate that: firstly, *PropSeg* provides a reproducible measurement of cord area both in healthy controls and people with MS, similarly to the widely used *AS* (Horsfield et al., 2010); secondly, *PropSeg* seems to be able to detect changes over time reliably, at least with the same sensitivity as the *AS* method; thirdly, *PropSeg* provides cord area measures that reflect physical disability, as shown by the presence of significant associations between obtained cord area values and physical disability, as well as being predictive of a specific measure of spinal cord dysfunction (ASIA-m) at one-year follow-up.

This current study demonstrates that a fully automated software package may be used to measure cord area in MS, acknowledging the fact that only T1-weighted MRI was used in this particular study; the use of any other type of contrast, or even the application of *PropSeg* to other neurological conditions merit investigation in their own right. As the software is automated, we chose to demonstrate its reproducibility by measuring the scan–rescan ICC, which was > 0.98 for two different segments of the cervical cord. This agrees strongly with the *AS* measurements obtained in this current study and a previous study, that also measured its reproducibility (Kearney et al., 2014a). Here, manual intervention was required to identify the vertebral levels (i.e. slices corresponding to C2/C3 and C2/C5), to ensure a direct comparison between the two segmentation methods. However, a new feature has recently been added to *PropSeg* that automatically identifies vertebral levels using template-based approaches, allowing the user to prespecify the cord segment(s) of interest (De Leener et al., in press). In conjunction with the probabilistic mapping of spinal levels based on vertebral levels (Cadotte et al., 2015), such information might provide more specific association between clinical deficits and the level of spinal cord atrophy, as shown in ALS (Cohen-Adad et al., 2013).

In order to use cord atrophy as an endpoint for a clinical trial the methodology must be sufficiently sensitive to a small reduction in cord area. In the current study we have shown that the reductions in cord area observed over one year, although not significant, were in line with the *AS* method, used as an anchor measure in this study. The lack of significant reduction may relate to the smaller number of patients followed up, which likely reduced the statistical power. The high CR and ES values obtained with *PropSeg* further emphasise the robustness of this technique when applied to a longitudinal study of cord atrophy in MS. However, the worse ES result observed at C2/C5 in all groups using *PropSeg* could be additionally informative. Bearing in mind the higher CR values of *PropSeg* as compared to *AS* at that level, coupled with the slightly lower ICC values observed for both methods at C2/C5 as compared to the C2/C3 level, this may be indicative of a reduced reliability of atrophy measurements when obtained at the C2/C5 level. The potential pitfalls of studying the C2/C5 level as opposed to C2/C3 have been mentioned elsewhere (Kearney et al., 2014a, Losseff et al., 1996, Reid, 1960) and the significance and relative clinical impact of the small variations in ICC, CR, and ES identified in this study have not been examined specifically. Nevertheless, it has been shown that, at least for the C2/C3 level, *PropSeg* provides reproducible measurements and can detect change with at least the same sensitivity as the *AS* method.

Owing to the longitudinal nature of this present study we were also able to examine the predictive ability of cord atrophy, in relation to physical disability in MS. The spinal cord specific measure of motor disability used in this study (ASIA-m) was predicted by cord atrophy using the fully automated *PropSeg* method. Importantly, as regards the univariable models, the obtained R-squared were generally at least as high in the *PropSeg* method models as in the *AS* method models, and several times higher in the clinical models using *PropSeg* measures. However, more commonly used scales of physical disability in MS (such as the EDSS and MSFC) were not predicted by either the *PropSeg* method or the *AS* method.

### 4.1 Limitations and future directions

A number of limitations should be considered when interpreting the results of this study. Firstly, *PropSeg* has been evaluated in MS using only T1-weighted images and therefore the performance of the method using other forms of contrast in MS will need to be investigated specifically. However, due to the time-efficient and fully automated nature of the method, such assessments may be easily carried out on retrospective data.

As previously mentioned, a subset only of the cohort included at baseline was followed up at one year. This could be addressed in a future longitudinal study, in which a greater number of the baseline participants are followed up. One factor that may facilitate such a study would be to include people with progressive MS that have lower levels of physical disability, so that with time severe disability does not become a prohibitive factor for scanning.

Furthermore, the followed-up cohort consisted of people with different subgroups of MS. This may have conceivably influenced the overall rate of atrophy observed, thereby influencing the predictive power of this MRI parameter. A future longitudinal study containing, either a single subgroup of MS, or a sufficiently large cohort, so that between group factors can be analysed would be of importance.

Lastly, the current study was performed on data acquired in a single centre. Although the results obtained were in line with the hypotheses being investigated, many clinical trials in MS are performed in multiple centres and these hypotheses have not been tested in such a scenario. It would therefore be of importance to determine the sensitivity to pathology when introducing different scanner manufacturers as confounder when using the *PropSeg* method. This could be addressed by analysing data from existing (or future) multi-centre trials in MS that include imaging of the spinal cord.

## 5 Conclusion

This study demonstrates that spinal cord atrophy may be measured reliably in multiple sclerosis using a fully automated image segmentation method. These results have direct implications for future clinical trials for neuroprotection in progressive MS, where previous attempts at spinal cord atrophy measurement have been limited by factors relating to reproducibility or sensitivity to change, both of which are addressed in this study.

## Figures and Tables

**Table 1:** Demographic and clinical characteristics of study participants at baseline.

	Controls n = 26	CIS n = 21	RRMS n = 26	SPMS n = 21	PPMS n = 26
Gender (F:M)	17:9	13:8	17:9	12:9	11:15
Mean age (± SD)	42 (10.5)	35 (9)	40 (10)	51 (10)	51 (9)
Mean disease duration (years/months for CIS)	N/A	5	7	19	10
Mean CSA (± SD) — *PropSeg* (C2/C3)	70.2 (7.4)	75.9 (7.9)	68.6 (7.7)	56.2 (10.1)	61.1 (9.3)
Mean CSA (± SD) — *AS* (C2/C3)	75.8 (7.7)	82.0 (8.2)	74.0 (7.3)	62.0 (10.5)	67.1 (10.6)
Mean CSA (± SD) — *PropSeg* (C2/C5)	72.4 (7.1)	77.9 (7.9)	71.3 (7.9)	58.2 (10.0)	62.5 (9.0)
Mean CSA (± SD) — *AS* (C2/C5)	78.7 (7.4)	84.7 (8.0)	77.5 (8.0)	64.4 (10.4)	69.8 (9.7)
Median EDSS (range)	N/A	1 (0–3.5)	3 (0–6.5)	7 (4.5–7.5)	6 (2–7)
Median TWT (range)	5 (4–6)	4.6 (3.4–9.8)	5.7 (3.4–9.6)	22.3 (5–180)	8.3 (5–180)
Median HPT (range)	18.9 (15.1–27.1)	20.7 (16.6–25.4)	20.5 (15–36.4)	29.6 (19.1–200.8)	28.9 (17.1–179.6)
Mean PASAT (± SD)	53 (5.3)	45.2 (9.4)	41.6 (14.6)	37 (19.2)	34.9 (18.8)
Median ASIA-m (range)	100 (–)	100 (98–100)	99 (74–100)	87 (63–98)	85 (54–100)
Median ASIA-s (range)	112 (–)	112 (84–112)	110 (98–112)	104 (84–112)	101.5 (90–112)

CIS: clinically isolated syndrome; RRMS: relapsing remitting MS; PPMS: primary progressive MS; SPMS: secondary progressive MS; EDSS: expanded disability status score; TWT: 25-foot timed walk test; HPT: 9-hole peg test; PASAT: 3 s paced auditory serial addition test B; ASIA: American Spinal Injury Association motor (m) and sensory (s) scores; SD: standard deviation.

**Table 2:** Intraclass correlation coefficient (ICC) for scan–rescan reliability using *PropSeg* and *AS* segmentation methods for measuring the cervical cord cross-sectional area (CSA) at the C2/C3 and C2/C5 levels in healthy control (n = 8) and MS cases (n = 8).

	ICC (95% CI)	
Healthy controls	C2/C3	C2/C5
CSA — *PropSeg*	0.992 (from 0.934 to 0.996)	0.990 (from 0.968 to 0.995)
CSA — *AS*	0.992 (from 0.977 to 0.995)	0.990 (from 0.973 to 0.994)
MS cases		
CSA — *PropSeg*	0.984 (from 0.938 to 0.991)	0.985 (from 0.734 to 0.991)
CSA — *AS*	0.992 (from 0.86 to 0.996)	0.994 (from 0.862 to 0.997)

ICC: intraclass correlation coefficient; CSA: cross-sectional area; CI: confidence interval.

**Table 3:** Ratio of change (CR) in CSA measures over one year.

	C2/C3		C2/C5	
Group	*PropSeg*	*AS*	*PropSeg*	*AS*
Controls	− 0.365	− 0.181	− 0.337	0.081
RRMS	− 0.296	− 0.116	− 0.357	− 0.217
SPMS	− 0.553	− 0.417	− 0.393	− 0.189
PPMS	− 0.744	− 0.883	− 0.179	− 0.205

RRMS: relapsing remitting MS; PPMS: primary progressive MS; SPMS: secondary progressive MS

**Table 4:** Effect size calculation.

	C2/C3		C2/C5	
Groups	*PropSeg*	*AS*	*PropSeg*	*AS*
RRMS (vs. controls)	− 0.2	− 0.042	− 0.195	− 0.26
SPMS (vs. controls)	− 0.209	− 0.171	0.067	− 0.319
PPMS (vs. controls)	− 0.6	− 0.707	− 0.014	− 0.292

RRMS: relapsing remitting MS; PPMS: primary progressive MS; SPMS: secondary progressive MS

**Table 5:** Associations between CSA measures and clinical scores at baseline (unadjusted).

	*PropSeg*		*AS*	
Regression coefficient (95% CI), p-value	R^2^	Regression coefficient (95% CI), p-value	R^2^
*C2/C3 measures*				
EDSS	− 0.13 (− 0.165 to − 0.094), p < 0.001	0.366	− 0.123 (− 0.157 to − 0.088), p < 0.001	0.347
TWT	− 1.652 (− 2.417 to − 0.886), p < 0.001	0.134	− 1.612 (− 2.354 to − 0.87), p < 0.001	0.136
HPT	− 1.136 (− 1.729 to − 0.543), p < 0.001	0.109	− 1.084 (− 1.660 to − 0.508), p < 0.001	0.105
PASAT	0.32 (0.059 to 0.581), p = 0.017	0.048	0.312 (0.059 to 0.566), p = 0.016	0.049
ASIA-m	0.533 (0.362 to 0.704), p < 0.001	0.244	0.492 (0.323 to 0.660), p < 0.001	0.22
ASIA-s	0.246 (0.133 to 0.359), p < 0.001	0.136	0.22 (0.109 to 0.331), p < 0.001	0.116

*C2/C5 measures*				
EDSS	− 0.128 (− 0.163 to − .093), p < 0.001	0.361	− 0.122 (− 0.157 to − 0.087), p < 0.001	0.341
TWT	− 1.759 (− 2.513 to − 1.003), p < 0.001	0.153	− 1.729 (− 2.467 to − 0.99), p < 0.001	0.154
HPT	− 1.153 (− 1.743 to − 0.562), p < 0.001	0.112	− 1.096 (− 1.675 to − 0.516), p < 0.001	0.106
PASAT	0.344 (0.084 to 0.603), p = 0.010	0.056	0.315 (0.061 to 0.57), p = 0.016	0.049
ASIA-m	0.526 (0.355 to 0.698), p < 0.001	0.239	0.497 (0.328 to 0.667), p < 0.001	0.223
ASIA-s	0.24 (0.127 to 0.353), p < 0.001	0.131	0.225 (0.114 to 0.336), p < 0.001	0.12

EDSS: expanded disability status score; TWT: 25-foot timed walk test; HPT: 9-hole peg test; PASAT: 3 s paced auditory serial addition test B; ASIA: American Spinal Injury Association motor (m) and sensory (s) scores; CI: confidence interval.

**Table 6:** Associations between CSA measures and clinical scores at one-year follow-up (unadjusted).

	*PropSeg*		*AS*	
Regression coefficient (95% CI), p-value	R^2^	Regression coefficient (95% CI), p-value	R^2^
*C2/C3 measures*				
EDSS	− 0.030 (− 0.01 to 0.039), p = 0.372	0.045	− 0.032 (− 0.09 to 0.028), p = 0.272	0.067
TWT	− 1.881 (− 4.23 to 0.466), p = 0.113	0.113	− 1.735 (− 3.917 to 0.447), p = 0.115	0.073
HPT	− 1.514 (− 3.168 to 0.14), p = 0.071	0.092	− 1.291 (− 2.842 to 0.26), p = 0.100	0.078
PASAT	0.216 (− 0.507 to 0.94), p = 0.547	0.011	0.147 (− 0.527 to 0.822), p = 0.660	0.006
ASIA-m	0.688 (0.287 to 1.09), p = 0.001	0.241	0.627 (0.246 to 1.01), p = 0.002	0.226
ASIA-s	0.23 (0.012 to 0.447), p = 0.039	0.107	0.207 (0.002 to 0.413), p = 0.048	0.099

*C2/C5 measures*				
EDSS	− 0.028 (− 0.093 to 0.038), p = 0.389	0.042	− 0.03 (− 0.091 to 0.03), p = 0.308	0.058
TWT	− 2.082 (− 4.376 to 0.213), p = 0.074	0.094	− 2.097 (− 4.26 to 0.066), p = 0.057	0.106
HPT	− 1.478 (− 3.097 to 0.141), p = 0.072	0.092	− 1.39 (− 2.91 to 0.136), p = 0.073	0.092
PASAT	0.234 (− 0.473 to 0.941), p = 0.505	0.013	0.182 (− 0.486 to 0.85), p = 0.584	0.009
ASIA-m	0.682 (0.287 to 1.077), p = 0.001	0.243	0.634 (0.257 to 1.01), p = 0.002	0.234
ASIA-s	0.255 (0.044 to 0.466), p = 0.019	0.137	0.228 (0.026 to 0.43), p = 0.028	0.121

EDSS: expanded disability status score; TWT: 25-foot timed walk test; HPT: 9-hole peg test; PASAT: 3 s paced auditory serial addition test B; ASIA: American Spinal Injury Association motor (m) and sensory (s) scores; CI: confidence interval.

**Table 7:** Independent predictors of clinical changes (unadjusted).

	*PropSeg*		*AS*	
Regression coefficient (95% CI), p-value	R^2^	Regression coefficient (95% CI), p-value	R^2^
*C2/C3 measures*				
EDSS	− 0.026 (− 0.099 to 0.047), p = 0.459	0.031	− 0.0302 (− 0.09 to 0.029), p = 0.298	0.06
TWT	− 1.888 (− 4.258 to 0.483), p = 0.115	0.074	− 1.493 (− 3.70 to 0.72), p = 0.178	0.054
HPT	− 1.491 (− 3.151 to 0.168), p = 0.077	0.089	− 1.163 (− 2.72 to 0.398), p = 0.139	0.063
PASAT	0.243 (− 0.481 to 0.966), p = 0.5	0.014	0.202 (− 0.47 to 0.874), p = 0.546	0.011
ASIA-m	0.7 (0.288 to 1.113), p = 0.001	0.237	0.602 (0.212 to 0.991), p = 0.003	0.204
ASIA-s	0.161 (− 0.07 to 0.390), p = 0.165	0.050	0.155 (− 0.057 to 0.367), p = 0.147	0.055

*C2/C5 measures*				
EDSS	− 0.024 (− 0.096 to 0.0492), p = 0.505	0.025	− 0.027 (− 0.087 to 0.034), p = 0.371	0.045
TWT	− 2.183 (− 4.503 to 0.137), p = 0.064	0.1	− 2.035 (− 4.199 to 0.13), p = 0.065	0.1
HPT	− 1.504 (− 3.142 to 0.132), p = 0.070	0.093	− 1.278 (− 2.796 to 0.239), p = 0.096	0.079
PASAT	0.239 (− 0.476 to 0.954), p = 0.501	0.013	0.173 (− 0.487 to 0.833), p = 0.597	0.008
ASIA-m	0.752 (0.358 to 1.145), p < 0.001	0.282	0.645 (0.27 to 1.02), p = 0.001	0.242
ASIA-s	0.203 (− 0.019 to 0.425), p = 0.073	0.082	0.192 (− 0.014 to 0.397), p = 0.066	0.086

EDSS: expanded disability status score; TWT: 25-foot timed walk test; HPT: 9-hole peg test; PASAT: 3 s paced auditory serial addition test B; ASIA: American Spinal Injury Association motor (m) and sensory (s) scores; CI: confidence interval.
